# Distinguishing Closely Related Amyloid Precursors Using an RNA Aptamer*

**DOI:** 10.1074/jbc.M114.595066

**Published:** 2014-08-06

**Authors:** Claire J. Sarell, Theodoros K. Karamanos, Simon J. White, David H. J. Bunka, Arnout P. Kalverda, Gary S. Thompson, Amy M. Barker, Peter G. Stockley, Sheena E. Radford

**Affiliations:** From the Astbury Centre for Structural Molecular Biology and School of Molecular and Cellular Biology, University of Leeds, Leeds LS2 9JT, United Kingdom

**Keywords:** Amyloid, Aptamer, Protein Aggregation, Protein Folding, Structural Biology, β_2_-Microglobulin, RNA Aptamer, Amyloid Fibril, Amyloid Precursor, Co-polymerization

## Abstract

Although amyloid fibrils assembled *in vitro* commonly involve a single protein, fibrils formed *in vivo* can contain multiple protein sequences. The amyloidogenic protein human β_2_-microglobulin (hβ_2_m) can co-polymerize with its N-terminally truncated variant (ΔN6) *in vitro* to form hetero-polymeric fibrils that differ from their homo-polymeric counterparts. Discrimination between the different assembly precursors, for example by binding of a biomolecule to one species in a mixture of conformers, offers an opportunity to alter the course of co-assembly and the properties of the fibrils formed. Here, using hβ_2_m and its amyloidogenic counterpart, ΔΝ6, we describe selection of a 2′F-modified RNA aptamer able to distinguish between these very similar proteins. SELEX with a N30 RNA pool yielded an aptamer (B6) that binds hβ_2_m with an EC_50_ of ∼200 nm. NMR spectroscopy was used to assign the ^1^H-^15^N HSQC spectrum of the B6-hβ_2_m complex, revealing that the aptamer binds to the face of hβ_2_m containing the A, B, E, and D β-strands. In contrast, binding of B6 to ΔN6 is weak and less specific. Kinetic analysis of the effect of B6 on co-polymerization of hβ_2_m and ΔN6 revealed that the aptamer alters the kinetics of co-polymerization of the two proteins. The results reveal the potential of RNA aptamers as tools for elucidating the mechanisms of co-assembly in amyloid formation and as reagents able to discriminate between very similar protein conformers with different amyloid propensity.

## Teaser

**Background:** Altering the co-polymerization of proteins into amyloid fibrils provides an opportunity for manipulating fibril assembly.

**Results:** NMR and kinetic analysis showed that an RNA aptamer distinguishes between two highly similar co-aggregating proteins.

**Conclusion:** RNA aptamers are specific and discriminatory probes able to modulate amyloid formation.

**Significance:** Aptamers can be used as tools to differentiate amyloid precursors that are closely related and alter assembly.

## Introduction

Despite the array of different proteins and peptides with distinct amino acid sequences that are known to be able to assemble into amyloid fibrils *in vitro* and/or *in vivo* (1), the precise molecular mechanism(s) by which these different proteins/peptides self-assemble into amyloid fibrils and how the assembly process results in disease remain unclear (2). Amyloid formation commences with the generation of aggregation-prone monomeric precursors. These species can be unfolded/disordered, partially structured or even native-like (3) and their structural properties, even though potentially similar to their non-amyloidogenic counterparts, dictate the fate of amyloid assembly (4). This is exemplified by the observation that the same amino acid sequence can form conformationally distinct amyloid structures *in vitro* by varying the temperature, altering the agitation conditions, adding co-solvents, metal ions or other molecules, or even changing the surface properties of the incubation vessel (reviewed in Ref. 5). An extra level of complexity is added by the ability of different protein/peptide precursors to co-polymerize, resulting in new fibril polymorphs with different amyloid architectures, stabilities, and/or different kinetics of assembly than those formed by each protein alone (4, 6, 7). Indeed, there are multiple examples of amyloidogenic proteins that are able to co-polymerize such as islet amyloid polypeptide and Aβ (6, 8), Tau and α-synuclein (9), and insulin and transthyretin (10). Although the importance of identifying and characterizing rarely populated amyloidogenic precursors is widely appreciated (3), this remains a significant challenge because of the transient nature and heterogeneity of assembly intermediates (11). The development of reagents able to discriminate aggregation-prone species among a pool of structurally similar molecules is crucial to deciphering the mechanisms of protein assembly into amyloid and to inform the design of therapeutic/diagnostic strategies able to target individual amyloid precursors (12).

**FIGURE 1.**
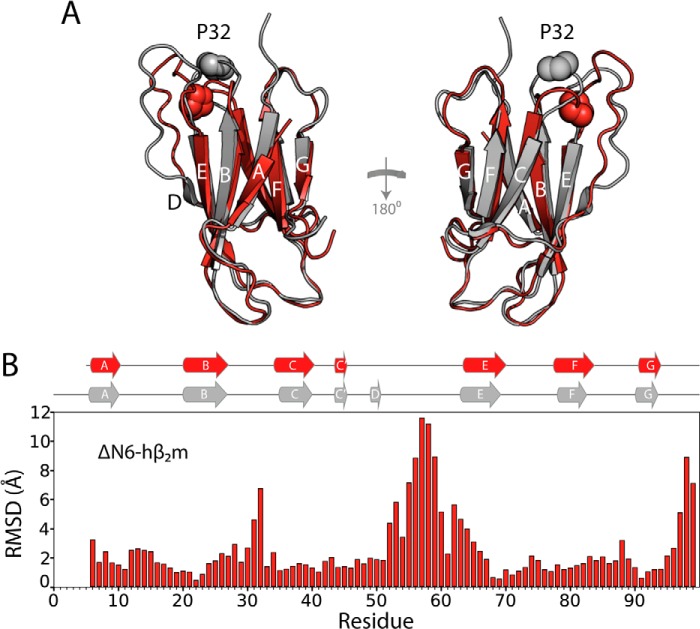
**Comparison of the structures of hβ_2_m and ΔΝ6.** *A*, the structure of hβ_2_m (*gray ribbon*, Protein Data bank code 2XKS (19)) and ΔN6 (*red* schematic, Protein Data Bank code 2XKU (19)). The two β-sheets of the proteins comprising the A, B, E, and D β-strands and the C, F, and G β-strands are shown. Pro-32 is shown in space fill. *B*, per residue r.m.s.d. chart for the backbone atoms of hβ_2_m and ΔΝ6 (overall backbone r.m.s.d. ∼ 1.5Å). The positions of the β-strands in these proteins are shown on *top* as *gray* (hβ_2_m) and *red* (ΔΝ6) ribbons.

Human β_2_-microglobulin (hβ_2_m)^5^ is a small protein that forms amyloid deposits in collagen-rich osteoarticular sites, resulting in the disorder dialysis-related amyloidosis (DRA) (13, 14). Despite the propensity of hβ_2_m to form amyloid fibrils *in vivo*, conditions that destabilize the native structure of hβ_2_m such as low pH (15), the presence of SDS (16), or other co-solvents or metal ions (17, 18), are required for fibril formation on an experimentally tractable time scale *in vitro*. Removal of the N-terminal six residues from hβ_2_m (the sequence IQRTPK), creating the variant ΔΝ6, disrupts the thermodynamic and kinetic stability of hβ_2_m and, as a result, ΔN6 can self-assemble into amyloid fibrils rapidly and spontaneously without the need to add detergents, metal ions, or other reagents (19, 20). ΔN6 retains a native-like structure, displaying a backbone r.m.s.d. of only ∼1.5 Å compared with hβ_2_m (19), and contains a non-native *trans* X-Pro-32 (see Fig. 1, *A* and *B*), which has been shown to be vital for fibril formation (21, 22). Isomerization of the X-Pro-32 bond results in structural reorganization of the side chains in the apical region of hβ_2_m, resulting in a protein with different surface hydrophobicity and electrostatic properties (19). ΔN6 can also promote the aggregation of hβ_2_m even when added in trace amounts (19), resulting in co-polymerization of both proteins into hetero-polymeric amyloid fibrils (4). This interaction allows amyloid formation of hβ_2_m to be investigated in the absence of additives at physiologically relevant pH values (4).

The design of molecules able to bind hβ_2_m or its amyloidogenic counterpart, ΔN6, would offer an opportunity to increase understanding of the interaction between these co-assembling monomers and explore the aggregation pathway that leads to their co-polymerization into amyloid fibrils. However, such a task is hindered by the high sequence and structural homology (see Fig. 1*A*) of the two proteins and their dynamic nature (19). In this study, we used *in vitro* selection to identify an RNA aptamer able to bind hβ_2_m preferentially to ΔN6 and to alter fibril co-assembly. Nucleic acid aptamer selection has been used previously to generate RNA aptamers able to discriminate monomeric PrP^SC^ and recombinant PrP^C^ (23, 24) and to bind to Aβ monomers rather than fibrils (25–27). Oligomers of amyloidogenic proteins have also been used as targets: DNA/RNA aptamers have been raised against oligomers of α-synuclein (28) and Αβ40 (29), respectively.

Previously, we used SELEX to isolate RNA aptamers against fibrillar hβ_2_m that were counter-selected against the low pH, partially unfolded, hβ_2_m monomer from which these fibrils were formed (30). Here, we extend this approach using SELEX to isolate 2′fluoro-modified RNA aptamers against native monomeric hβ_2_m. The selected aptamer discriminates in its binding to hβ_2_m or ΔN6 at pH 6.2, conditions in which both proteins are folded, but only ΔN6 is able to assemble spontaneously into amyloid fibrils (19). The hβ_2_m specific aptamer was minimized to a 44-nucleotide-long fragment and its binding interface, affinity, and specificity for hβ_2_m were determined. The aptamer binds tightly and specifically to the β-sheet of hβ_2_m containing the A, B, E, and D β-strands, but only weakly and less specifically to ΔN6. Addition of the aptamer to a mixture of hβ_2_m and ΔN6 under conditions (pH 6.2) that promote co-assembly (4) disfavors the interaction between the two proteins early in assembly, making hβ_2_m remain soluble for longer. The results reveal the ability of RNA aptamers to discriminate and bind to a specific protein conformer within a complex mixture of structurally similar co-polymerizing species, altering the course of amyloid assembly.

## EXPERIMENTAL PROCEDURES

### 

#### 

##### Protein Preparation

hβ_2_m and ΔN6 were expressed and purified as described previously (19). For NMR experiments, ^15^N- and ^13^C-labeled hβ_2_m and ΔN6 were prepared as described in Ref. 31.

##### Biotinylation and Immobilization of hβ_2_m

Monomeric hβ_2_m (∼1 mg) was biotinylated (EZLink^TM^ Sulfo-NHS-LC-LC-biotin, Pierce Biotechnologies) at pH 7 using a 20-fold molar excess of biotin over the total protein concentration, according to the manufacturer's instructions. The biotinylated monomer was then immobilized on 1-μm streptavidin-coated microspheres (Dynabeads^TM^, Invitrogen) using the manufacturer's instructions.

##### In Vitro Selection

A Biomek 2000 laboratory automation work station (Beckman Coulter) was used to perform 12 rounds of *in vitro* selections with an N30 library of 2′F-modified pyrimidine RNA, encompassing ∼10^15^ potential sequences, and transcribed using the Y639F/H784A variant of T7 RNA polymerase (32), using minor modifications of the protocols described previously (30). Selections were carried out in 50 mm MES buffer containing 120 mm NaCl, pH 6.2. Negative selections were carried out at each round of SELEX using streptavidin Dynabeads coated with Tris-inactivated linker. Stringency was increased after round 5 by decreasing the number of beads containing monomeric hβ_2_m by half and increasing the number of washes from 10 to 13. The reverse transcriptase-PCR products were analyzed by native PAGE after each group of five rounds of selection to confirm the isolation of products for the next round of selection. Individual aptamer clones were produced by *in vitro* transcription using 10 mm final concentrations of each nucleotide triphosphate using 2′F-CTP and 2′F-UTP for production of modified RNAs. RNA concentrations were determined using the following extinction coefficients: B6, 1026.3 mm^−1^ cm^−1^; B6 minimized (B6min), 553.2 mm^−1^ cm^−1^; B9, 1054.2 mm^−1^ cm^−1^.

##### Synthesis of Minimized B6

B6min (5′-GGG AAU UCU GAG CUA CUC CCU UUU GGG CCC GGC UAU GAU UCC CG-3′) was synthesized with and without 2′F-modified pyrimidine nucleotides (named 2′F B6min and 2′OH B6min, respectively) on an ABI 394 RNA synthesizer at a 1 μm scale using the protocols described previously (33). The phosphoramidites used for synthesis of 2′F-B6min were as follows: *N*-benzoyl-protected adenosine, *N*-dimethylformamidinyl-protected guanosine, *N*-acetyl-protected-2′-fluoro deoxycytidine, and 2′-fluoro-deoxyuridine. For synthesis of 2′-OH B6min *N*-acetyl-protected-2′-fluoro-deoxycytidine and 2′-fluoro-deoxyuridine were replaced with *N*-acetyl-protected-cytidine and uridine phosphoramidites (Link Technologies, Ltd.). Cyanoethyl-(*N*,*N*′-diisopropyl) and *t*-butyldimethylsilyl groups were present on the 3′- and 2′-hydroxyl groups. Treatment with ammonia-saturated methanol at room temperature for 24 h was used to remove protecting groups and to cleave RNA from controlled pore glass resin. Methanol was removed under vacuum and the RNA pellet re-suspended in anhydrous dimethyl sulfoxide. One volume of triethylamine trihydrofluoride was added and incubated at room temperature to remove *t*-butyldimethylsilyl, the deprotected RNA was precipitated with butan-1-ol and resuspended in diethylpyrocarbonate-treated water (Severn Biotech) before being purified by reverse-phase HPLC at 55 °C (34). RNA fractions were collected, lyophilized, and desalted into 18.2 megohm H_2_O. The RNA was analyzed on a 10% (w/v) denaturing polyacrylamide urea gel stained with ethidium bromide. The RNA was synthesized using *N*-dimethylformamidinyl-protected guanosine controlled pore glass to avoid incorporation of a pyrimidine with a ribose sugar at the 3′-end. This additional guanosine has no effect on the secondary structure of 2′F-B6min or 2′-OH B6min as predicted by Mfold (35).

##### Surface Plasmon Resonance

A BIAcore3000 instrument was used with a streptavidin-coated gold sensorchip (BIAcore SA chip). A flow-rate of 10 μl min^−1^ was used with a running buffer of 50 mm MES, 120 mm NaCl, pH 6.2. 50 μl of 50 μg ml^−1^ of biotinylated monomer was injected over separate flow cells so that ∼200 response units of protein was immobilized. RNAs were dialyzed into running buffer before injection across the surface to minimize bulk refractive index effects. Flow cells were regenerated using a 20-μl wash of 5 m NaCl. All sensorgrams were corrected by subtracting the signals of an equivalent injection across an underivatized flow cell. Data were analyzed using the manufacturer's software (BIAevaluation).

##### Intrinsic Fluorescence Quenching

The fluorescence of tryptophan residues in 1 μm hβ_2_m or ΔN6 was excited at 290 nm, and fluorescence emission was measured between 300 and 390 nm in the presence of increasing concentrations of 2′F B6min or 2′OH B6min in 50 mm MES buffer containing 120 mm NaCl, pH 6.2, at 25 °C. Due to the large extinction coefficient of the RNA aptamer at 260 nm (553.2 mm^−1^ cm^−1^), the absorbance of the hβ_2_m/aptamer solution at 290 nm was measured after each addition of aptamer to ensure that the absorbance of the solution was below 0.05 at 290 nm so that inner filter effects do not contribute to the data (36). Fluorescence emission was measured using a Photon Technology International QM-1 spectrofluorimeter using 10-nm slit-widths. The data for binding of 2′F B6min to hβ_2_m were normalized to a value of 0 in the absence of aptamer and a fluorescence signal of 1 obtained upon saturation. The data were then fitted to the following logistic equation to extract the half maximal effective concentration (EC_50_) using in-house scripts,

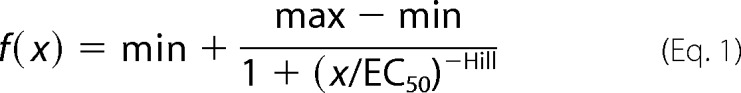
 where max and min represent the maximum and minimum fluorescence signals, respectively, Hill is the Hill coefficient, *f*(*x*) is the fluorescence units, and *x* is the concentration of the aptamer in nm. For 2′F B6min to ΔΝ6 and 2′OH B6min to hβ_2_m, no change in fluorescence was observed over the concentration range studied.

##### NMR Spectroscopy

Samples of ^13^C-^15^N-labeled protein (60 μm) in 50 mm MES buffer containing 120 mm NaCl, pH 6.2, 0.02% (w/v) sodium azide, 0.1 mm EDTA, 90% (v/v) H_2_O/10% (v/v) D_2_O were used for NMR experiments. Synthetic 2′F B6min or 2′OH B6min was added into the protein solution from a concentrated stock (typically 200 μm). Working at a concentration of 60 μm necessitated the use of a sensitivity optimized strategy for obtaining assignments. This was achieved using a reduced dimensionality approach based on Hadamard encoding (37). Sequential assignments were obtained from analysis of Hadamard encoded two-dimensional H(N-H_2_)CA and H(N-H_2_)(CO)CA experiments where a two-step Hadamard matrix is introduced on ^15^N to subdivide the peaks into two subspectra where most signals can be addressed from their ^1^H shift alone and the dimensionality can be reduced to 2 to maximize sensitivity. Spectra were recorded at 25 °C on a Varian Inova 750 MHz spectrometer equipped with a cryogenic probe and were processed using NMRPipe and analyzed using Collaborative Computational Project for NMR analysis (38). To calculate the intensity profiles shown in Fig. 7, peak intensities were normalized to the number of scans and the protein concentration used for each experiment. Intensity profiles were calculated as the ratio of the normalized peak intensity of each resonance in the apo spectrum (*I*_0_) *versus* the normalized intensity at the same position but in the aptamer-bound spectrum (*I*). Therefore, the loss of native signal plotted in Fig. 7 does not require full assignment of the aptamer-bound spectrum.

##### Assembly of Amyloid Fibrils

40 μm hβ_2_m and 40 μm ΔN6 in the presence or absence of two molar equivalents of 2′F B6min were co-incubated in 50 mm MES buffer containing 120 mm NaCl, pH 6.2, 0.02% (w/w) sodium azide at 600 rpm, 37 °C in a Thriller Thermoshaker incubator (Peqlab). Each sample (100 μl) was incubated in 0.5-ml plastic Eppendorf tubes. Aliquots of 8 μl were removed at different time points during incubation and immediately centrifuged at 14,000 × *g* for 20 min. The supernatant was separated from the pellet, and both supernatant and pellet were frozen at −20 °C for subsequent analysis by SDS-PAGE.

##### SDS-PAGE

The effect of 2′F B6min on fibril formation was monitored using 15% polyacrylamide Tris-Tricine gels. Samples of the supernatant and pellet were thawed, and the pellet was resuspended in 8 μl of 50 mm MES buffer containing 120 mm NaCl, pH 6.2. Both the supernatant and resuspended pellet were added 1:1 to loading buffer (50 mm Tris-HCl, pH 6.8, 100 mm DTT, 2% (w/v) SDS, 0.1% (w/v) bromphenol blue, and 10% (v/v) glycerol) and boiled for 5 min before loading 15 μl into the gel. Gels were stained with Coomassie Instant Blue (Expedeon) and imaged by SnapGene software (Syngene).

##### Electron Microscopy

At the end of fibril assembly, 10 μl of sample were applied to a carbon-coated grid. The grid was then carefully dried with filter paper before it was negatively stained by the addition of 10 μl of 4% (w/v) uranyl acetate as described in Ref. 39. Micrographs were recorded on a Philips CM10 or a JEOL JEM-1400 electron microscope.

## RESULTS

### 

#### 

##### Selection of hβ_2_m-specific 2′F-RNA Aptamers

**FIGURE 2.**
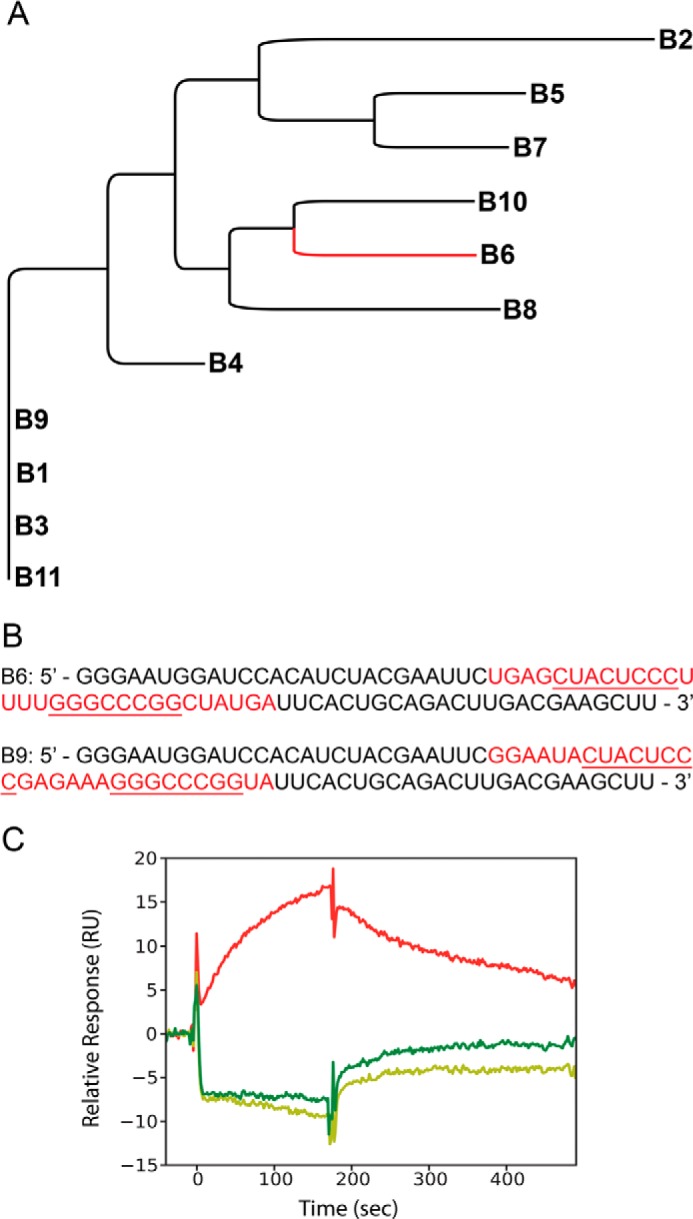
**Aptamer selection.** *A*, the relationship of B6 to the 10 other sequences from the SELEX pool. *B*, sequences of aptamers B6 and B9. The selected regions are shown in *red*, and their common sequence motifs are *underlined. C*, surface plasmon resonance traces generated upon incubation of 1 μm 2′F B6 (50 mm MES buffer, 120 mm NaCl, pH 6.2) over flow cells immobilized with hβ_2_m (*red*), ΔN6 (*dark green*), or murine β_2_m (*light green*). *RU*, response units.

Co-incubation of ΔN6 and hβ_2_m results in the two proteins polymerizing into hetero-polymeric amyloid-like fibrils that are morphologically and thermodynamically distinct compared with fibrils formed by ΔN6 or hβ_2_m alone (4). To control the co-assembly of these proteins, we attempted to select RNA aptamers capable of discriminating between natively folded hβ_2_m and ΔN6 at pH 6.2 (Fig. 1*A*). Hβ_2_m was biotinylated (predominantly at the N terminus and on Lys-7 and/or Lys-92) and immobilized as a target on streptavidin-coated magnetic beads, as described previously (30). The initial SELEX protocol used an N30 2′F-pyrimidine substituted RNA library to create aptamers resistant to nucleases (25, 32) and included counter-selection against ΔN6 monomers immobilized as for hβ_2_m, as well as long straight and worm-like amyloid fibrils formed from hβ_2_m at acidic pH (40). This protocol resulted in the removal of most of the aptamers from the selected pool, consistent with the different protein conformers containing many epitopes in common. We therefore abandoned counter-selections, except against biotin linker-blocked streptavidin beads alone. In addition, in the final round of SELEX, aptamers bound to bead-immobilized hβ_2_m were competed off the beads using non-biotinylated hβ_2_m in solution to ensure that the selected aptamer pool contained ligands for native epitopes, SELEX was carried out at pH 6.2 as this is both optimal for hβ_2_m/ΔN6 co-polymerization (hβ_2_m does not self-assemble spontaneously on a relevant time scale at this pH, whereas ΔN6 assembles rapidly) (4) and is physiologically relevant for amyloid deposition in patients with DRA (41, 42). In total, 12 SELEX rounds were performed, with rounds 6–12 having increased stringency (see “Experimental Procedures”). From the final pool, 11 RNA clones were sequenced and aligned using the program AliBee (43). These were then clustered using the phylogenetic software Clustal Omega (Fig. 2*A*) (44).

##### Isolation of 2′F B6 and Characterization of the Binding Affinity to hβ_2_m

**FIGURE 3.**
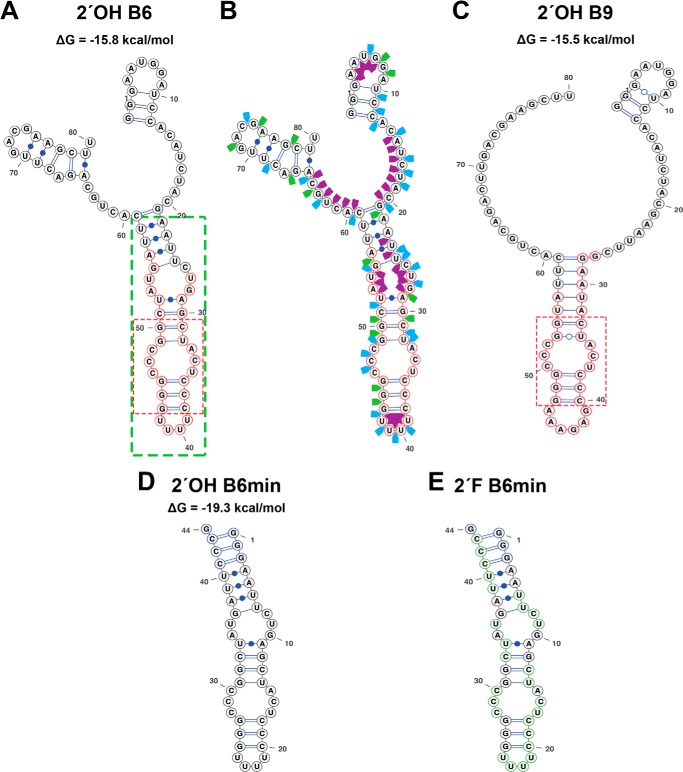
**Secondary structures of the B6 and B9 aptamers.** *A*, the Mfold secondary structure prediction of the full-length B6 aptamer with the nucleotides within the *green box* showing the region truncated to create the B6min aptamer sequence. Nucleotides *circled* in *red* define the random region. *B*, enzymatic solution structure probing of the full-length B6 transcript with the random region highlighted in *red*. Cleavage sites by the G-specific RNase T1 (*green arrows*), U and C-specific RNase A (*blue arrows*), and single-stranded RNA specific S1 nuclease (*purple arrows*) are shown. *C*, the Mfold of the full-length B9 aptamer with the selected region highlighted as described in *A*. The *dotted red boxes* in *A* and *C* showed the conserved sequences and secondary structure elements of both aptamers. *D*, secondary structure of 2′ OH B6min. *E*, 2′F B6min stem loops. These have additional 5′-GGG and 3′-CCCG sequences added to increase their folded stability. 2′F pyrimidines are circled in *green* in *E*.

Two aptamers, B6 and B9, contained the most frequently occurring sequence motifs within the sequenced clones and showed some motif similarities (Fig. 2, *A* and *B*). To identify which aptamer to utilize for further studies, an initial binding assay was employed using surface plasmon resonance. Biotinylated hβ_2_m, ΔN6, or the non-amyloidogenic murine β_2_m (45) were immobilized on separate flow cells and aptamer binding monitored at pH 6.2. 2′F B6 binds to hβ_2_m with an apparent affinity of ∼500 nm (*red trace* in Fig. 2*C*), but did not bind ΔN6 (*dark green trace*) or murine β_2_m (*light green trace*). In contrast, binding of 2′F B9 was so weak that the *K_d_* could not be determined (data not shown). The secondary structure of B6, computed via Mfold to be a stable stem loop (ΔG° ∼ −16 kcal/mol) (Fig. 3*A*) (35), was confirmed using enzymatic solution structure probing (Fig. 3*B*). This analysis suggests that the selected region consists of an extended base-paired stem loop interrupted by several single-stranded bulges with a terminal loop consisting of a poly-U tetraloop (highlighted in *red* in Figs. 2*B* and 3*A*). Note that both Mfold and enzymatic probing were of transcripts containing natural pyrimidines. B9 is predicted to have several equivalently stable structures that are all identical in the selected region, which forms a structure very similar to that of B6 around one of the bulges (Fig. 3*C*). B9 differs radically at the terminal loop, however, which is composed of six purine nucleotides. It appears that the loop is the motif that provides much of the binding energy for the interaction of B6 with hβ_2_m. Further characterization was therefore restricted to B6 and its derivatives.

**FIGURE 4.**
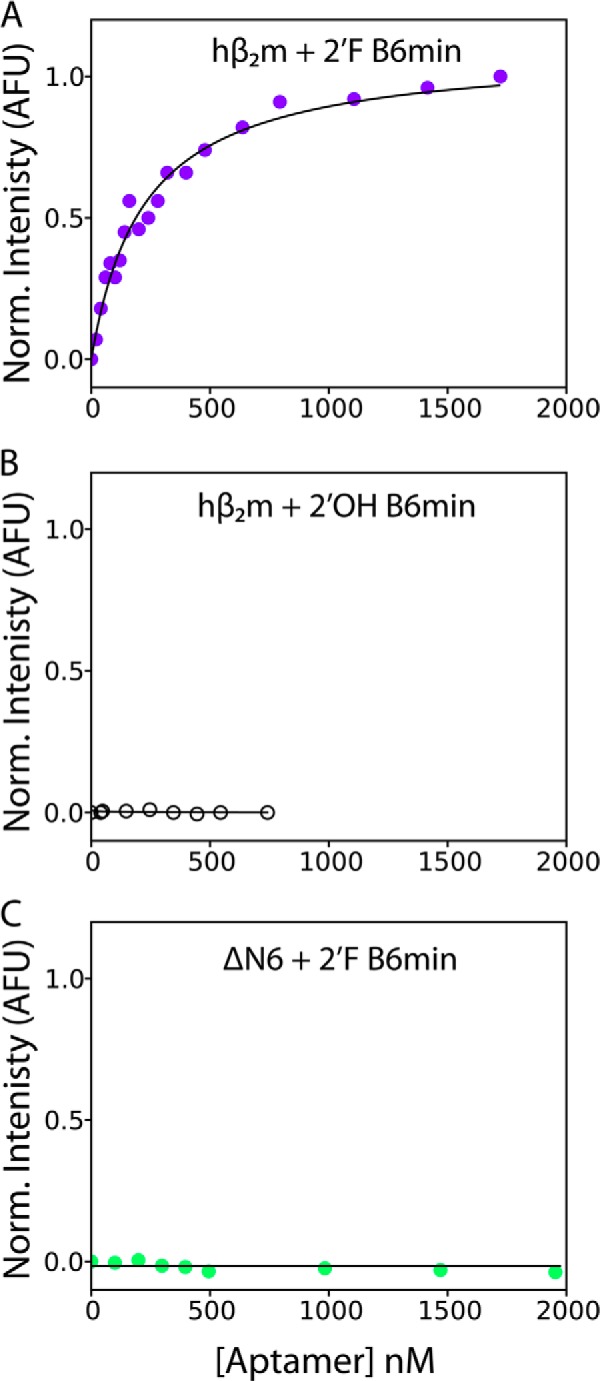
**Binding of 2′OH B6min and 2′F B6min to hβ_2_m and** Δ**N6 measured using intrinsic tryptophan fluorescence.** *A*, normalized tryptophan fluorescence of hβ_2_m (1 μm) upon addition of 2′F B6min (0–1.7 μm). The data are fitted to a logistic equation (*solid line*). The data are normalized (*Norm.*) between 0 (no aptamer) and 1 (the fluorescence signal in the presence of 1.7 μm aptamer) (see “Experimental Procedures”). *B*, titration of hβ_2_m (1 μm) with 2′OH B6min. *C*, titration of ΔN6 (1 μm) with 2′F B6min. No fluorescence change was observed over the concentrations of aptamer added in *B* and *C*. These data were normalized between 0 (no aptamer) and 1 (the fluorescence signal when 1.7 μΜ of 2′F B6min was added to hβ_2_m. All experiments were performed in 50 mm MES buffer, 120 mm NaCl, pH 6.2. *AFU*, arbitrary fluoresence units.

A truncated 44-nucleotide version of the 110-nucleotide full-length B6 was produced encompassing nucleotides 22 to 59 with 2′OH (termed 2′OH B6min) (Fig. 3*D*) or 2′F pyrimidines (termed 2′F B6min) (Fig. 3*E*), *i.e.* all of the selected region defining the stem loop with some stabilizing additional base pairs. We examined the solution binding of 2′F B6min to native hβ_2_m and ΔN6 using fluorescence spectroscopy. hβ_2_m has two tryptophan residues: Trp-60, which lies in the DE loop (Fig. 1*A*) and is solvent exposed, and Trp-95, which lies toward the C terminus of the 100-residue protein and is buried. Tryptophan fluorescence of hβ_2_m can be used to probe changes in conformation or chemical environment upon aptamer binding, with Trp-95 reporting on alterations within the hydrophobic core (46), whereas Trp-60 is sensitive to ligand binding (at least in proximity to this residue) at the protein surface. The fluorescence emission spectrum of monomeric hβ_2_m (1 μm) was monitored upon titration with 2′F B6min. The results showed a decrease in tryptophan emission intensity (with little change in λ_max_), consistent with binding of 2′F B6min to the protein surface adjacent to Trp-60. Fitting the normalized intensity of Trp fluorescence *versus* the concentration of 2′F B6min added (Fig. 4*A*) (see “Experimental Procedures”) yielded a Hill slope of 0.99 ± 0.06, suggesting a specific one-site binding event, with an EC_50_ of 223 ± 10 nm. Similar assays using 2′OH B6min showed no binding to hβ_2_m (Fig. 4*B*), indicating that the 2′F modifications to the pyrimidines are required for tight binding, consistent with the contribution of the polyU tetraloop to affinity. The fluorescence assay also showed no binding of 2′F B6min to ΔN6 monomers (Fig. 4*C*), consistent with the surface plasmon resonance data with full-length aptamers. These results indicate, therefore, that 2′F B6min is capable of discriminating between hβ_2_m and ΔN6. 2′-Fluoro-ribose is known to prefer different sugar pucker conformations compared with unmodified residues (O4′-*endo versus* C3′-*endo*, respectively (47)). This could alter the conformation of the tetraloop and hence its interaction with the protein.

##### Determining the Binding Interface of B6min with Natively Folded hβ_2_m Using NMR

**FIGURE 5.**
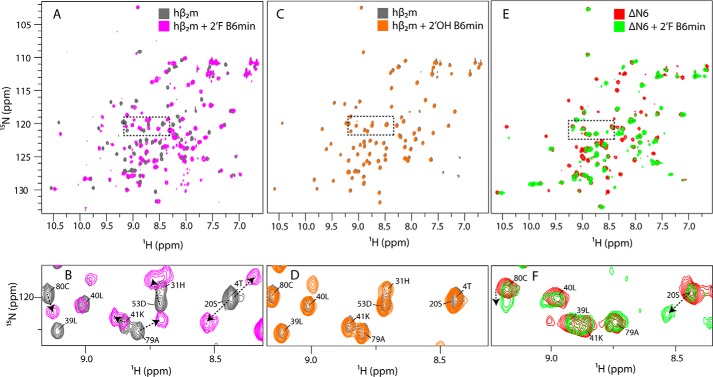
**Chemical shift changes upon the addition of aptamers to hβ_2_m and ΔN6.** *A*, the ^1^H-^15^N HSQC spectrum of ^15^N,^13^C-labeled hβ_2_m (60 μm) alone (*gray*) or in the presence of two molar equivalents of 2′F B6min (*magenta*). *B*, expansion of the region *boxed* in *A. C*, the ^1^H-^15^N HSQC spectrum of ^15^N,^13^C-labeled hβ_2_m (60 μm) alone (*gray*) or in the presence of two molar equivalents of 2′OH B6min (*orange*). *D*, expansion of the region *boxed* in *C. E*, the ^1^H-^15^N HSQC spectrum of ^15^N,^13^C-labeled ΔN6 (60 μm) alone (*red*) or in the presence of two molar equivalents of 2′F B6min (*green*). *F*, expansion of the region *boxed* in *E*. Chemical shift changes in *B*, *D*, and *F* are annotated with *arrows*. All spectra were obtained at 25 °C, pH 6.2.

**FIGURE 6.**
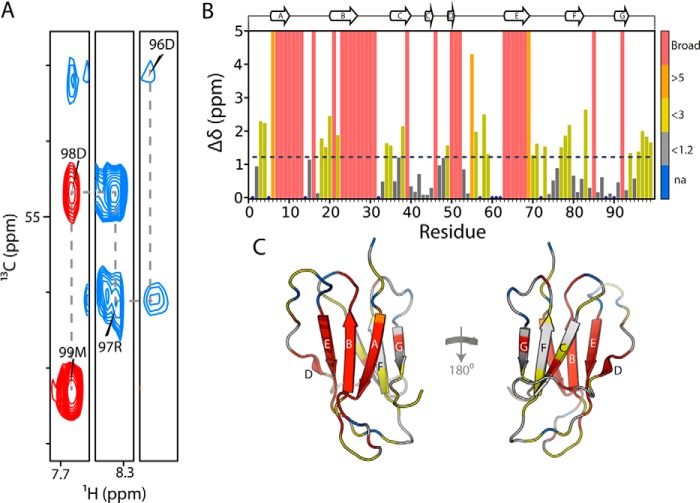
**Chemical shift changes upon binding of 2′F B6min to hβ_2_m.** *A*, zoomed in regions of the two-dimensional HNCA spectrum of ^13^C,^15^N-hβ_2_m with 2 molar equivalents of 2′F B6min. The assignment walk on the Cα values is shown for the four residues. *B*, chemical shift changes of hβ_2_m upon interaction with 2′F B6min. Total chemical shift change was calculated as √((5 × Δδ ^1^H)^2^ + (Δδ ^15^N)^2^). Residues for which assignments were not possible as a consequence of exchange broadening or large chemical shift perturbation are given an arbitrary value of 5 ppm and are shown in *red*. The *dashed line* represents two S.D. over the entire data set. *C*, the structure of hβ_2_m colored according to the measured chemical shift changes shown in *B*.

To determine whether binding of 2′F B6 to hβ_2_m induces conformational changes in the protein and to map the binding site in residue-specific detail, 2′F B6min was titrated into ^15^N,^13^C-labeled hβ_2_m at 0, 0.25, 0.5, 1.0, and 2.0 molar equivalents at pH 6.2, and ^1^H-^15^N HSQC spectra were recorded. The ^1^H-^15^N HSQC spectrum of the 2:1 mixture of 2′F B6min and hβ_2_m is shown in Fig. 5, *A* and *B*. Addition of 2′F B6min results in the appearance of new peaks in the spectrum and the loss of resonances assigned to native apo-hβ_2_m, indicating that the complex is in slow exchange with the apoprotein, as expected for a high affinity complex. The chemical shift changes involve some, but not all, resonances, indicative of binding of the aptamer to a specific surface. The ^1^H-^15^N HSQC spectrum of the hβ_2_m-2′F B6min complex was assigned using a combination of two-dimensional and three-dimensional NMR techniques (see “Experimental Procedures”) (Fig. 6*A*). The low sample concentration (60 μm) and relatively large size of the complex (25.6 kDa) made assignment challenging. Of the 88 main chain resonances in the ^1^H-^15^N HSQC spectrum of hβ_2_m, 55 were successfully assigned. The assigned spectrum of the 2′F B6min-hβ_2_m complex was then used to map the binding site for 2′F B6min on the surface of the protein. Residues with the largest chemical shift differences upon aptamer binding are located on the face of hβ_2_m that contains the A, B, E, and D β-strands (Fig. 6, *B* and *C*). A significant number of residues in this region could not be assigned unambiguously in the spectrum of the complex, suggesting that they experience large chemical shift differences upon aptamer binding or are not detected due to exchange line broadening (Fig. 6, *B* and *C*). The titration was also performed using 2′OH B6min (Fig. 5, *C* and *D*). No changes in the chemical shifts of hβ_2_m were observed, even at the 2:1 aptamer/hβ_2_m molar ratio, confirming that the presence of 2′F-modified pyrimidines is vital for high affinity binding. To investigate whether 2′F B6min is able to recognize ΔΝ6, 2 molar equivalents of the aptamer were added to 60 μm ^15^N-labeled ΔΝ6, and binding was again assessed by monitoring changes in chemical shifts (Fig. 5, *E* and *F*). In this sample, the large changes in chemical shifts observed previously in the hβ_2_m-2′F B6min complex (Fig. 5, *A* and *B*) were not detected (*e.g.* compare residues Lys-41 and Ala-79 in Fig. 5, *B* and *F*). For some resonances, small changes in chemical shift were observed; however, in those cases, the chemical shifts did not saturate, even in the presence of a 2-fold molar excess of 2′F B6 (*e.g.* residues Ser-20 and Cys-80 (Fig. 5*F*)). The results thus confirm a significantly lower affinity of this aptamer for ΔN6.

**FIGURE 7.**
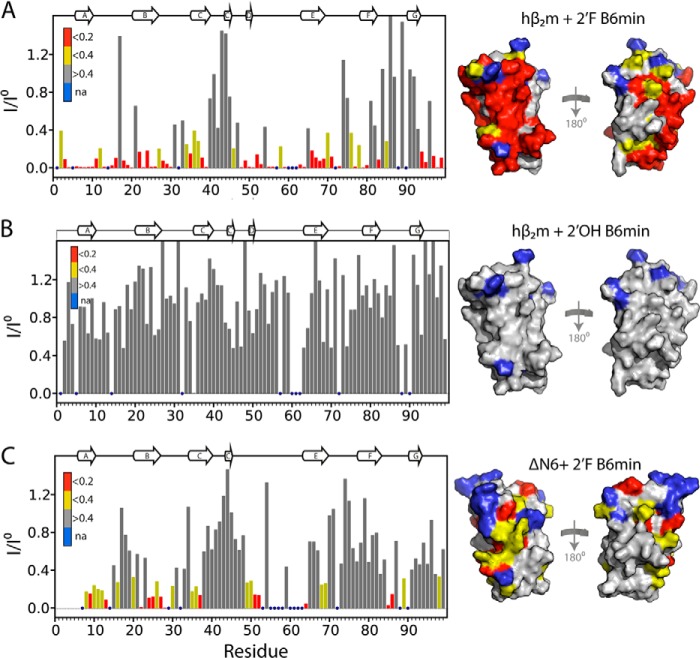
**2′F B6 distinguishes between two highly similar proteins.** *A*, plot of the loss of signal intensity of resonances in native hβ_2_m upon binding to a 2-fold molar excess of 2′F B6min using data shown in Fig. 5*A*. Profiles were calculated as the ratio of the peak intensity in the presence (*I*) or absence (*I*^o^) of a 2-fold molar excess of aptamer. Intensity profiles were normalized to residues 40–45 that are not involved in the interface. Residues with a ratio of <0.2 are colored *red*, those showing a ratio between 0.2 and 0.4 are colored *yellow*, and those with no significant decrease in intensity are colored *gray*. The structure of hβ_2_m drawn as a surface representation is shown on the *right* color-coded using the same scale. Residues with no assignments (*na*) are shown in *blue. B*, as described in *A*, but for the interaction of 2′OH B6min and hβ_2_m. *C*, as described in *A*, but for the interaction of 2′F Β6min with ΔΝ6. The secondary structure elements of the proteins are shown as ribbons on *top* of the panels.

To obtain more detailed information about the position of the 2′F B6min binding site on the surface of hβ_2_m, the intensity of each resonance was determined in the presence of a 2-fold molar excess of aptamer and compared with the intensity of its apo counterpart. The results of this analysis are shown in Fig. 7*A*. Resonances arising from residues in the A, B, E, and D β-strands, the AB and DE loops, residues 3–6 in the N-terminal region, and the C-terminal 6 residues (*red* in Fig. 7*A*) lose >80% of their intensity in the spectrum of the complex. These residues form a contiguous surface on hβ_2_m (Fig. 7*A*) and include the N-terminal 6 residues of hβ_2_m that are lacking in ΔN6 and confer increased affinity, consistent with these residues forming part of the interface between the RNA and the protein. Consistent with this, there is little or no change in intensity for residues that lie in the CC′ loop and F and G β-strands on the opposite face of hβ_2_m (*gray* in Fig. 7*A*). By contrast with these results, addition of a 2-fold molar excess of 2′OH B6min to hβ_2_m has no significant effect on the intensities of the resonances of native hβ_2_m (Fig. 7*B*), consistent with its lack of binding.

A similar analysis was performed to assess the possible interaction between 2′F B6min and ΔΝ6. As expected based on the fluorescence titration results shown in Fig. 4*C*, little change in intensity was observed for the vast majority of residues in this sample (compare Fig. 7, *A* and *C*), consistent with weak binding to ΔN6. Furthermore, the residues that do show a difference in resonance intensity differ from those involved in the 2′F B6min-hβ_2_m interface. For example, although resonances belonging to residues in the AB loop, the E strand, and the C-terminal 6 residues of native hβ_2_m diminish in intensity by >80% upon interaction with 2′F B6min, these resonances are largely unaffected (retaining >60% average intensity) when ΔΝ6 is incubated with the aptamer. Moreover, the residues in ΔN6 showing the largest decrease in intensity upon addition of 2′F B6min (*red* in Fig. 7*C*) are spread throughout the structure of the protein, suggesting that binding of 2′F B6min to ΔΝ6 is less specific than the 2′F B6-hβ_2_m interaction. These differences in binding presumably explain the insensitivity of tryptophan fluorescence observed upon addition of 2′F B6min to ΔΝ6.

**FIGURE 8.**
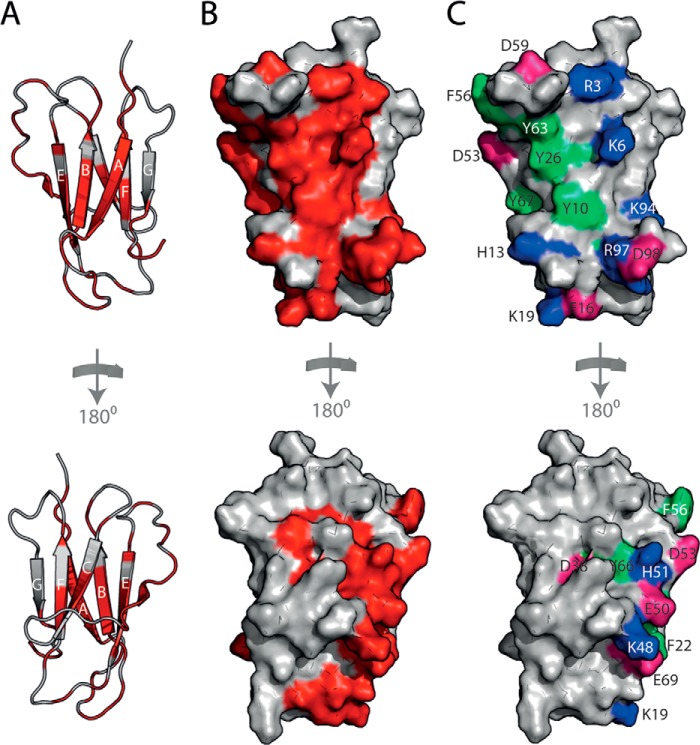
**Mapping the 2′F B6min-hβ_2_m binding site.** *A*, the residues in hβ_2_m that show the largest decrease in intensity upon interaction with 2′F B6min are shown in *red* on the structure of hβ_2_m (*gray* schematic) and predominantly involve residues in the A, B, E, and D β-strands of hβ_2_m. By contrast, the C, F, and G β-strands show relatively little change in intensity (*bottom*). *B*, surface representation of hβ_2_m highlighting the interface residues (*red*). *C*, the 2′F B6min-hβ_2_m binding interface involves seven aromatic residues (*light green*), seven positively charged residues (*blue*), and seven negatively charged residues (*pink*).

The 2′F B6min-hβ_2_m interface defined by these experiments (Fig. 8, *A–C*) includes a large number of aromatic side chains (Tyr-10, Phe-22, Tyr-26, Phe-56, Tyr-63, Tyr-66, and Tyr-67, *green* in Fig. 8*C*), as often found in protein-RNA complexes (48). The residues involved in the binding interface might also be expected to be positively charged, but there appears to be an equal balance of positively charged residues (Arg-3, Lys-6, His-13, Lys-19, Lys-48, His-51, and Lys-94 (*blue* in Fig. 8*C*)) and negatively charged side chains (Glu-16, Asp-38, Glu-50, Asp-53, Asp-59, Glu-69, and Asp-98 (*pink* in Fig. 8*C*)). Analysis of the NMR data shows that residues 3–6 are clearly part of the binding site. This sequence is absent in ΔΝ6, which binds very poorly, and contains two positive charges (Arg-3 and Lys-6), but no negative charges. This region is therefore a candidate for a favorable electrostatic interaction with the aptamer. Indeed, murine β_2_m, which does not bind this aptamer (Fig. 2*C*), has a Gln substituted for Lys at residue 6 (N-terminal sequence of murine β_2_m is IQKTPQ), implying that Lys-6 is a likely key recognition element for hβ_2_m.

##### 2′F B6min Alters the Co-assembly of ΔN6 and hβ_2_m

**FIGURE 9.**
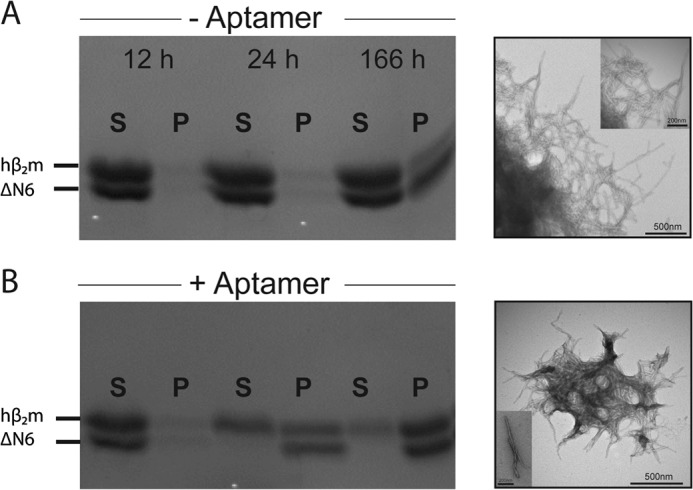
**2′F B6min affects hβ_2_m-ΔN6 co-polymerization into fibrils.** *A*, the course of aggregation of mixtures of hβ_2_m and ΔN6 (each 40 μm) in the absence of a 2-molar excess of aptamer determined by SDS-PAGE. The morphology of the aggregates formed after 166 h is shown by TEM. *B*, as described for *A* but in the presence of a 2-fold molar excess of 2′F B6min. *S*, supernatant; *P*, pellet. Incubation was performed in 50 mm MES, 120 mm NaCl, pH 6.2, with 600 rpm agitation at 37 °C. The *scale bars* on the TEM images represent 500 nm. For the *inset*, *scale bars* in the TEM images are 200 nm.

The NMR and fluorescence data presented above indicate that 2′F B6min binds tightly to hβ_2_m, but only weakly and non-specifically to ΔN6. At pH 6.2 hβ_2_m does not self-assemble into amyloid fibrils *in vitro* over a time scale of several weeks at a concentration of 40 μm, even using significant agitation (13, 49, 50). In contrast, ΔN6 rapidly and quantitatively forms fibrils under these conditions (19, 49). When the two proteins are incubated together at this pH, they co-polymerize, forming hetero-polymeric fibrils with distinct structural properties compared with either of their homo-polymeric counterparts (4). To determine whether 2′F B6min is able to affect the co-aggregation of hβ_2_m and ΔN6 (due to preferential binding of the aptamer to one of the fibrillating monomers), the two proteins were mixed (each at a concentration of 40 μm) in the presence or absence of a 2-fold molar excess of 2′F B6min (160 μm) at pH 6.2. Assembly was monitored by separating soluble and insoluble material by centrifugation and subsequent analysis of each fraction by SDS-PAGE (see “Experimental Procedures”) (Fig. 9). In parallel, a sample of the assembly products were monitored using transmission EM (TEM) to confirm whether amyloid fibrils were produced. The results of these experiments showed that in the absence of 2′F B6min, each protein remains in the soluble fraction up to the 24-h time point, after which time insoluble material containing both proteins forms (Fig. 9*A*). After 166 h of incubation, both proteins are also found in the pellet presumably due to their co-polymerization into fibrils (4). By contrast, in the presence of 2′F B6min aggregation occurs more rapidly, with >90% of ΔN6 and ∼40% of hβ_2_m forming fibrillar material after 24 h. TEM images of the samples after 166 h confirmed that the insoluble material in the pellets contains amyloid fibrils (Fig. 9, *A* and *B*), although the precise location of each protein within each fibril (*i.e.* the extent to which co-polymerization occurred) could not be ascertained from these experiments. Presumably, the interaction between soluble ΔN6 and hβ_2_m is inhibited by 2′F B6min, leading to rapid polymerization of ΔN6, which in part co-polymerizes with hβ_2_m.

## DISCUSSION

**FIGURE 10.**
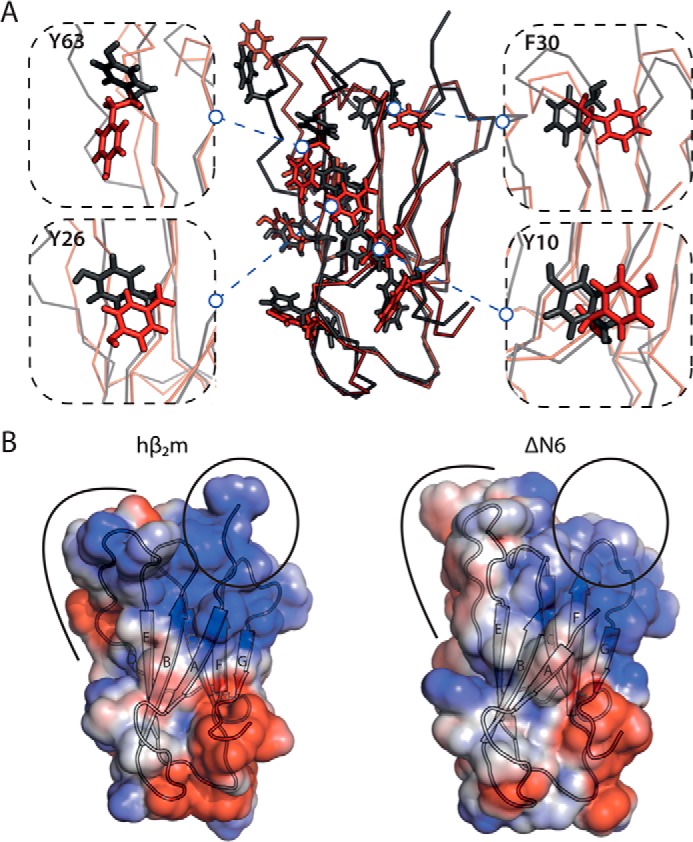
**Structural differences between hβ_2_m and ΔΝ6 in the aptamer binding surface.** *A*, the aromatic residues located in the interface between 2′F B6min and hβ_2_m (see Fig. 8) are highlighted as sticks on hβ_2_m (*black ribbon*) and ΔΝ6 (*red ribbon*). Close ups of four residues are shown alongside. *B*, the structure of hβ_2_m (*left*) and ΔΝ6 (*right*) shown as a surface representation colored by its electrostatic potential (*blue*, positive; *red*, negative). The N-terminal region is highlighted in a *circle*, and the DE loop region is annotated with a *black arc*.

To derive a structural mechanism of amyloid formation, the identity and structure of all assembling components must be defined, and how these species interact and form the cross-β-structure of amyloid should be determined. Here, RNA SELEX has been used to generate a specific, high-affinity aptamer (2′F B6) against monomeric hβ_2_m. Importantly, despite only subtle differences in the structures of monomeric hβ_2_m and its N-terminal truncation variant ΔN6 at pH 6.2 (Fig. 1, *A* and *B*), 2′F B6 is able to discriminate between these structures, showing tight and highly specific binding to the β-sheet containing the A, B, E, and D strands of hβ_2_m. By contrast, weak, nonspecific binding is observed to ΔN6 that is detectable only at the high protein and RNA concentrations used for NMR (60 μm protein). The discrimination between hβ_2_m and ΔN6 by 2′F B6 can be explained, at least in part, by the presence of Lys-6 in the binding interface. However, given that the binding interface appears to involve an extended region spanning the A, B, E, and D β-strands, other residues must also contribute to affinity. Indeed, differences in the organization of residues on the surfaces of hβ_2_m and ΔN6 that result from the isomerization of the X-Pro-32 peptide bond from the native *cis* isomer in hβ_2_m to the *trans* isomer in ΔN6 (19), and/or the decreased stability (13) and increased conformational dynamics of ΔN6 compared with hβ_2_m (19), may also contribute to 2′F B6 discriminating between these otherwise similar structures. For example, although the structure of the backbone is highly conserved between hβ_2_m and ΔN6 (Fig. 1*B*), the orientation of the side chains of aromatic residues involved in the aptamer binding interface differs significantly (Fig. 10*A*). Furthermore, the organization of hydrophobic and charged residues on the surface formed by the A, B, E, and D β-strands in hβ_2_m differs significantly from ΔN6 (Fig. 10*B*). Accordingly, the apical region of this surface in hβ_2_m is more highly positively charged than its equivalent in ΔN6 (this region contains the N-terminal six amino acids, including Lys-6) (Fig. 10*B*). In addition, the organization of negatively charged residues (involving the AB loop, the EF loop, and the C terminus) also differs between the two proteins (Fig. 10*B*). In total, therefore, the balance between electrostatic and hydrophobic residues, crucial for nucleic acid binding (51), is distinct in hβ_2_m and ΔΝ6, partly due to the removal of the N-terminal six amino acids, and due in part to differences in solvent exposure of hydrophobic residues in the DE and BC loops in the two proteins that occur as a consequence of X-Pro-32 isomerization.

The role of ΔN6 in DRA is not currently understood. Although ΔN6 is present in the amyloid deposits found in patients with DRA (52), it remains unknown whether the N-terminal truncation of hβ_2_m occurs pre- or post-fibril formation. Additionally, the interaction between hβ_2_m and ΔN6 *in vitro* is complex, with ΔN6 possessing the ability to convert monomeric hβ_2_m into an amyloidogenic conformation (4, 19, 53) and to act as a fibrillar seed able to be elongated with hβ_2_m monomers (19, 49). The aptamer selected here may be useful as an analytical probe to derive greater clarity in understanding the early stages of hβ_2_m and ΔN6 co-assembly into amyloid. Given the complexity of amyloid formation, where self-assembly can be initiated by one or more rare conformers that may differ subtly in structure, and that different oligomeric species may exhibit profoundly different cytotoxicity (54, 55), RNA aptamers offer unique potentials as reagents for the analysis of, and interference with, amyloid formation.

The specific and tight binding of 2′F B6 to hβ_2_m alters the course of amyloid assembly in mixtures of hβ_2_m and ΔN6 at pH 6.2. Thus, aptamer binding to hβ_2_m disfavors the incorporation of hβ_2_m into amyloid fibrils during co-assembly with ΔN6 and results in more rapid fibril formation. In the presence of the aptamer, hβ_2_m molecules will become incorporated into fibrils only after aptamer dissociation, possibly by cross-seeding with preformed ΔN6 fibrils (4, 49). Alternatively, ΔN6 may promote conversion of hβ_2_m to an amyloidogenic conformation once 2′F B6min dissociates (4, 19, 53), pulling the equilibrium toward co-assembly into fibrils. Given that amyloid formation is under kinetic control, the development of aptamers able to bind their targets with slow off-rates (even for the same apparent *K_d_*) would provide an effective strategy to control assembly. Such aptamers could be isolated by increasing the length of time of the elution steps in SELEX as stringency is increased. Alternatively, coupling of the RNA aptamer to a molecule with known affinity to the target could provide a route to achieving this goal by exploiting avidity effects. Doxycycline, a small molecule tetracycline analog, has been shown to modulate the formation of hβ_2_m fibrils *in vitro* (56), to reduce articular pain and improve movement in DRA patients (57), and to correct a locomotory defect in *Caenorhabditis elegans* expressing hβ_2_m (58). Analysis of the hβ_2_m-doxycycline complex using NMR suggests that the highest affinity binding site (IC_50_ ∼ 50 μm (56)) involves residues that lie in the C-terminal region of strand A, the N-terminal region of strand B, and the central residues of the AB loop (56). A second, lower affinity binding site involves the N-terminal region and residues in the BC and DE loops. An intriguing possibility, therefore, would be to create an aptamer linked to doxycycline such that the relatively tight and specific binding of 2′F B6 can be exploited to enhance binding of doxycycline to its target interface. Creation of such bipartite molecules has been shown to be a highly effective strategy, not just for enhancing the effectiveness of RNA aptamers as delivery vehicles (59–61), but in many other applications (62–64).

In conclusion, the biophysical and biochemical studies presented here demonstrate that RNA aptamers can be highly specific and discriminatory probes, modulating co-polymerization reactions and controlling the course of amyloid assembly. How the 2′F B6-hβ_2_m complex changes as fibril formation proceeds and the effect of the aptamer on hetero-polymorphic fibril structure and stability will require further studies, for example, by exploiting the powers of solid-state NMR to analyze fibril structures (65, 66). Further characterization and modification of 2′F B6 will potentially allow the affinity of the aptamer for hβ_2_m to be increased, and selection of aptamers specific for ΔN6 will also allow detailed biophysical analysis of the role of ΔN6 in hβ_2_m-ΔN6 co-polymerization. Understanding this process further may shed light on the molecular mechanisms of fibril formation and how the protein precursors of hetero-polymeric assemblies can be modulated to tailor the extent, rate, and structure of amyloid fibrils.

## Footnotes

5 The abbreviations used are:

hβ_2_m: human β_2_-microglobulin
TEM: transmission electron microscopy
r.m.s.d.: root-mean-square deviation
SELEX: systematic evolution of ligands by exponential enrichment
DRA: dialysis-related amyloidosis.
